# Differing epidemiological dynamics of Chikungunya virus in the Americas during the 2014-2015 epidemic

**DOI:** 10.1371/journal.pntd.0006670

**Published:** 2018-07-30

**Authors:** Yi Tan, Brett E. Pickett, Susmita Shrivastava, Lionel Gresh, Angel Balmaseda, Paolo Amedeo, Lihui Hu, Vinita Puri, Nadia B. Fedorova, Rebecca A. Halpin, Matthew P. LaPointe, Marshall R. Cone, Lea Heberlein-Larson, Laura D. Kramer, Alexander T. Ciota, Aubree Gordon, Reed S. Shabman, Suman R. Das, Eva Harris

**Affiliations:** 1 J. Craig Venter Institute, Rockville, Maryland, United States of America; 2 Sustainable Sciences Institute, Managua, Nicaragua; 3 Laboratorio Nacional de Virología, Centro Nacional de Diagnóstico y Referencia, Ministry of Health, Managua, Nicaragua; 4 Florida Department of Health, Bureau of Public Health Laboratories, Tampa, Florida, United States of America; 5 Wadsworth Center, New York State Department of Health, Albany, New York, United States of America; 6 Department of Epidemiology, University of Michigan, Ann Arbor, Michigan, United States of America; 7 Division of Infectious Diseases and Vaccinology, School of Public Health, University of California, Berkeley, Berkeley, California, United States of America; Louisiana State University, UNITED STATES

## Abstract

Chikungunya virus (CHIKV) has been detected sporadically since the 1950s and includes three distinct co-circulating genotypes. In late 2013, the Asian genotype of CHIKV was responsible for the Caribbean outbreak (CO) that rapidly became an epidemic throughout the Americas. There is a limited understanding of the molecular evolution of CHIKV in the Americas during this epidemic. We sequenced 185 complete CHIKV genomes collected mainly from Nicaragua in Central America and Florida in the United States during the 2014–2015 Caribbean/Americas epidemic. Our comprehensive phylogenetic analyses estimated the epidemic history of the Asian genotype and the recent Caribbean outbreak (CO) clade, revealed considerable genetic diversity within the CO clade, and described different epidemiological dynamics of CHIKV in the Americas. Specifically, we identified multiple introductions in both Nicaragua and Florida, with rapid local spread of viruses in Nicaragua but limited autochthonous transmission in Florida in the US. Our phylogenetic analysis also showed phylogeographic clustering of the CO clade. In addition, we identified the significant amino acid substitutions that were observed across the entire Asian genotype during its evolution and examined amino acid changes that were specific to the CO clade. Deep sequencing analysis identified specific minor variants present in clinical specimens below-consensus levels. Finally, we investigated the association between viral phylogeny and geographic/clinical metadata in Nicaragua. To date, this study represents the largest single collection of CHIKV complete genomes during the Caribbean/Americas epidemic and significantly expands our understanding of the emergence and evolution of CHIKV CO clade in the Americas.

## Author summary

Chikungunya is an arboviral disease that causes fever and acute viral febrile illness, which may be followed by months or years of debilitating joint pain in humans. There are currently no vaccines or anti-viral drugs to prevent or treat chikungunya virus (CHIKV) infection. In late 2013, CHIKV was introduced into the Caribbean and spread rapidly throughout the Americas. CHIKV infections in the United States were primarily reported in Florida, with sporadic cases of traveler-associated infection reported in other states. However, little is known about the molecular evolution of the virus during this epidemic. Here, we sequenced a large number of CHIKV strains from Nicaragua, Florida and New York. Despite multiple introductions, limited local transmission was documented in Florida; in contrast, in Nicaragua, rapid local dissemination was observed. This study greatly increases the number of publicly-available CHIKV complete genome sequences from the Americas and provides a more comprehensive insight into the evolution of CHIKV during a geographically-diverse epidemic. This may have important implications for the control and prevention of other mosquito-borne viruses in the Americas, such as Zika, dengue, and West Nile viruses.

## Introduction

Chikungunya virus (CHIKV) belongs to the *Alphavirus* genus in the *Togaviridae* family and is an arthropod-borne virus spread by *Aedes* mosquitoes. CHIKV has a positive-sense single-stranded RNA genome that is 11.8 kb in length and encodes both a nonstructural and a structural polyprotein that are separated by a subgenomic promoter. Although CHIKV causes an acute febrile illness with clinical manifestations similar to other co-circulating mosquito-borne viral diseases, such as dengue, CHIKV infection may be followed by months or years of sequelae including rheumatic disease and debilitating joint pain [1].

The first reported outbreak of chikungunya occurred in eastern Africa during 1952–1953 [2–4]. CHIKV was detected sporadically in limited regions in Africa and Asia until the early 2000s [5–8]. Three distinct genotypes of CHIKV have been identified: West African, Asian, and East/Central/South African (ECSA) [9]. In 2005, the CHIKV ECSA genotype reemerged and caused multiple explosive epidemics across Africa, Asia, and the Indian Ocean, which then subsided [10–13]. In late 2013, the CHIKV Asian genotype was introduced into the Americas and rapidly spread through numerous Caribbean, South American, and Central American countries via *Aedes aegypti* mosquitoes [14–16].

There is a growing interest in understanding the epidemiology and molecular evolution of CHIKV during the 2014–2015 epidemic in different regions of the Americas. In Nicaragua, the first imported case was reported in July 2014, and autochthonous cases were reported since September 2014 [17]. The introduction of CHIKV into Nicaragua was followed by a small epidemic wave in 2014–2015 and a much larger second wave in 2015–2016. In North America, chikungunya infections were reported in travelers returning from Latin America and the Caribbean, with limited autochthonous transmission of CHIKV reported in Florida in July 2014 [18]. Following our recent effort of complete genome sequencing of CHIKV from the Americas [19], we strategically collected and sequenced another 185 complete genomes using next-generation sequencing methods representing multiple geographic locations, especially Nicaragua in Central America and Florida in the United States, during the 2014–2015 Caribbean/Americas epidemic. Here we reconstructed the evolutionary history of the CHIKV Asian genotype and described the different epidemiological dynamics of CHIKV in Nicaragua and Florida. We also investigated the association between CHIKV sequence variation with clinical features of the infected patients. Furthermore, we determined the statistically significant amino acid substitutions of CHIKV from the Caribbean/Americas epidemic, as well as assessed minor variants and intra-host diversity that were observed in these strains through deep-sequencing. To our knowledge, this dataset represents the largest single collection of CHIKV complete genomes from the Caribbean/Americas epidemic and augments our understanding of the emergence and evolution of CHIKV in the Americas.

## Materials and methods

### Summary of samples in this study and virus isolation

This study was based on three collections of CHIKV samples:

#### Nicaragua

To study the introduction and clinical spectrum of CHIKV in Nicaraguan children, two studies of chikungunya were established as extensions of an existing NIH-funded hospital-based study of dengue and an ongoing community-based cohort study of dengue and influenza, which are conducted in close collaboration with the Nicaraguan Ministry of Health. Samples for sequencing were collected during the first and second waves of the epidemic in 2014–15 and 2015–16, respectively. In addition, samples collected from a chikungunya household transmission study that enrolled children and adults were included. Finally, samples from the Ministry of Health National Surveillance System were also included in the collection [17]. Here, we determined the complete genome sequence for 108 CHIKV-positive samples from the above three studies and the national surveillance system during 2014 to 2015, with an additional strain collected in January 2016.

#### Returning travelers from the Caribbean to Florida

In 2014, Florida experienced a large increase in cases of chikungunya in travelers returning primarily from the Caribbean. The Florida Bureau of Public Health Laboratories (BPHL) confirmed 122 of these imported cases and all 12 locally transmitted cases from May 2014 to December 2014. Archived samples from a subset of these cases were used in this study. Here, a total of 67 complete genomes were sequenced and included in the current analysis, from travelers returning from the Caribbean and several cases of autochthonous transmission in Florida.

#### Returning travelers from the Caribbean and Asia to New York

We sequenced 5 CHIKV isolates from travelers returning from the Dominican Republic in 2014 and 5 historical samples spanning the time period from 1995–2008 that were obtained from the New York State Department of Health (NYSDOH).

Each collection used distinct protocols to isolate viral genetic material for sequencing. NYSDOH: Vero cell cultures were inoculated with CHIKV-positive sera, then total RNA was extracted from supernatants using MagMax Total Viral Isolation kit and shipped to the J. Craig Venter Institute (JCVI). BPHL: Sera from positive CHIKV cases either underwent RNA extraction directly from the original serum or from cell culture inoculation prior to shipment. Nicaragua: Total RNA from CHIKV-positive sera or first-passage isolates in C6/36 cells was extracted using the Qiagen QIAamp Viral RNA Mini Kit and shipped to JCVI.

### Next-generation sequencing, genome assembly, and annotation

Viral RNA, which was shipped to the JCVI from each of the participating institutions, was subjected to sequence-independent single-primer amplification (SISPA) [20]. In cases where sequence-independent amplification failed, a sequence-specific overlapping primer amplification strategy was used to generate the entire genome of CHIKV. The primer sequences used to amplify the required regions in each sample are reported in the respective GenBank sequence records. Barcoded libraries were pooled and adaptors were ligated following the manufacturer's instructions (Illumina). Libraries were sequenced on the Illumina MiSeq (2x300 bp Paired End (PE)) instrument.

After sequencing, barcoded reads from each sample were deconvoluted and trimmed to eliminate low-quality regions, SISPA hexamer primers and barcode sequences. Trimmed reads were subjected to *de novo* assembly with the *clc_novo_assemble* program in the CLC Bio software suite. The resulting contigs were queried against a custom full-length CHIKV reference database to determine the closest reference. Sequences were then mapped to the selected reference for each sample using the *clc_ref_assemble_long* program in the CLC Bio suite. A final mapping of all next-generation sequences to the updated reference sequences was performed with CLC Bio’s *clc_ref_assemble_long* program. When sufficient read data were available, reference sequences were extended to capture as much of the untranslated regions (UTRs) as possible. Assemblies were manually validated and curated prior to annotation with VIGOR [21]. VIGOR was used to predict genes and detect any potential sequencing errors. The annotation was subjected to manual inspection and quality control before submission to GenBank. All sequences generated as part of this study were submitted to GenBank as part of Bioproject PRJNA294670.

### Phylogenetic analyses

We combined the newly-determined 185 complete CHIKV genome sequences together with 271 CHIKV genome sequences available in GenBank (http://www.ncbi.nlm.nih.gov/genbank/, as of October 1, 2016) and generated a dataset with a total of 456 complete genome sequences of CHIKV. We excluded vaccine strains, synthetic recombinant strains, and other non-wildtype sequences. Because of the large number of insertion/deletion regions in the UTRs, these regions were excluded from the phylogenetic analyses (456_CDS). Sequences were aligned using the MUSCLE program in MEGA 6.0 with manual adjustment [22]. Potential recombination in the 456_CDS dataset was screened for using seven methods (RDP, GENECONV, Chimaera, MaxChi, SiScan, 3Seq, and BootScan) implemented in the Recombination Detection Program version 4.46 (RDP4) [23]. Sequences having p-values less than 1×10^−4^ in at least two methods were reported as evidence of recombination, and the one predicted recombinant strain (KJ679578) was removed from further phylogenetic analyses. Nucleotide and amino acid similarities across complete CDS and in each gene were also calculated. The individual amino acid sites under diversifying selection were detected by three different codon-based maximum likelihood methods: single most likely ancestral reconstruction (SLAC), fixed effects likelihood (FEL), and IFEL (similar to FEL, but selection is only tested for along internal branches of the phylogeny) using the Datamonkey webserver (www.Datamonkey.org) [24–26]. A significance cutoff of p<0.05 was used for all methods.

A maximum likelihood (ML) phylogenetic tree of complete coding sequences of CHIKV was inferred in MEGA 6.0 [27]. A general time reversal (GTR) nucleotide substitution model with a gamma distribution of among-site rate variation and a proportion of invariable sites (GTR+Γ+I) was selected as the best-fit model by Modeltest in MEGA 6.0 and used in the tree inference. The robustness of the ML tree was assessed by bootstrap analyses of 1000 pseudo-replicates, and the phylogenetic tree was midpoint rooted.

Due to the long branches of three genotypes (historical West African, ECSA, and Asian) suggested by our previous ML phylogeny and the very limited number of sequences in the West African genotype (n = 11), the phylogenetic trees of the ECSA and Asian genotypes were separately reconstructed using the Bayesian Markov Chain Monte Carlo (BMCMC) method available in the BEAST V1.8.2 package (http://tree.bio.ed.ac.uk/software/BEAST) [28]. Two sequences from the ECSA genotype, STMWG01/2011 and Angola/m2022/1962, were excluded from the BEAST analysis because of potential laboratory contamination or mutations that occurred during passaging [19]. Maximum likelihood phylogenies for the ECSA dataset and Asian dataset were also constructed using the methods described above and rooted by the oldest sequence in the dataset. To incorporate temporal information, each sequence in both datasets was assigned a date of origin that corresponded to the time of collection. To better estimate the spatial diffusion of the viruses, sequences were grouped by the different regions where they were collected. For 442 (out of 456) sequences with detailed travel history information, we assigned the regions based on the country of origin (Table 1). In the discrete phylogeographic analyses, the GTR+Γ+I substitution model selected by Modeltest in MEGA 6.0 was used under a relaxed molecular clock (uncorrelated lognormal, UCLN). The demographic model, Gaussian Markov Random Fields (GMRF) Bayesian skyride coalescent tree prior was employed in the BEAST runs. In each BEAST run, to ensure convergence of effective sampling size parameters (ESS) over 200, a Markov chain Monte Carlo (MCMC) was run for 500 million generations, and a 10% burn-in was removed. The results were assessed using the Tracer program v1.6.0 (http://tree.bio.ed.ac.uk/software/tracer) to ensure that convergence was achieved. The posterior distribution of BMCMC trees was summarized as the maximum clade credibility (MCC) tree and generated using TreeAnnotator v1.8.2 (available in BEAST v1.8.2 package), with the first 10% of trees removed as burn-in. MCC trees were visualized using FigTree v1.4.3 (http://tree.bio.ed.ac.uk/software/FigTree).

**Table 1:** Regions of sampling countries^a^.

Genotype	Region	Sampling country	No. of samples
ECSA	CAfrica: Central Africa	Central African Republic, Democratic Republic of the Congo, Gabon	6
		
	EAfrica: East Africa	Kenya, Tanzania, Uganda	7
		
	EAsia: East Asia	China, Japan	20
		
	Euro: Europe	France, Italy	6
		
	IndiaO: Indian Ocean	Comoros, Madagascar, Mauritius, Mayotte, La Reunion island	15
		
	MLSEA: Mainland Southeast Asia	Thailand, Cambodia, Myanmar	30
		
	MTSEA: Maritime Southeast Asia	Indonesia, Malaysia, Singapore	16
		
	SAfrica: South Africa	South Africa, Angola	3
		
	SAmerica: South America	Brazil	4
		
	SAsia: South Asia	India, Sri Lanka, Bangladesh	63
		
	WAfrica: West Africa	Senegal	1
		
	WAsia: West Asia	Yemen	1
Asian	CAmerica: Central America	Nicaragua, EI Salvador, Guatemala, Honduras, Panama	120
		
	NAmerica: North America	USA, Mexico	17
		
	SAmerica: South America	Brazil, Colombia, Guyana	10
		
	CariB: Caribbean	Saint Barthelemy, Dominican Republic, Haiti, Jamaica, Saint Lucia, Martinique, Puerto Rico, British Virgin Islands, US Virgin Islands, Trinidad and Tobago	84
		
	PaciO: Pacific Ocean	Micronesia, New Caledonia, French Polynesia, Samoa	7
		
	EAsia: East Asia	China	2
		
	SAsia: South Asia	India	5
		
	MLSEA: Mainland Southeast Asia	Thailand	6
		
	MTSEA: Maritime Southeast Asia	Indonesia, Malaysia, Philippines	19

^a^Regions were assigned based on the country of origin for 442 sequences with detailed travel history information.

### Association between virus phylogeny, geographic, demographic, and clinical features of CHIKV-infected patients

Host demographic information was available for all Nicaraguan samples, and clinical information was available for 95 of 108 of the Nicaraguan samples. To determine whether there was phylogenetic clustering by age or sex of the patient, virus sequences were grouped into 4 classes according to host age (<5 years, 5–10 years, 11–18 years, and >18 years old) or into 2 classes by sex respectively. Similarly, each patient was classified as either positive or negative for each clinical symptom individually. The clinical symptoms analyzed included history of: dehydration, headache, vomiting, myalgia, arthralgia, fever, rash, abdominal pain, and retro-orbital pain. The strength of association between the phenotypic features described above and the CHIKV phylogeny was determined using two phylogeny-trait association statistics, the parsimony score (PS) and the association index (AI) tests, both of which were implemented in the Bayesian tip association significance testing (BaTS) program [29]. A significance cutoff level of p<0.05 was used in both statistics. A null distribution of these statistics was determined using the posterior distribution of Nicaragua-only phylogenetic trees obtained from the BEAST package described above.

The posterior distribution of Caribbean-only phylogenetic trees generated by BEAST was also used to assess the strength of geographic clustering in the data by BaTS. Each sequence in the Caribbean clade was therefore assigned to a character state by its sampling country. For the sequences collected in North America with detailed travel history information, we assigned the states based on the travel history.

### Meta-CATS and structural analysis

The coding regions for the nonstructural and structural polyprotein coding regions were extracted from the same nucleotide-based multiple sequence alignments that were used for the phylogenetic tree reconstruction. Each of the coding regions was then translated into amino acid sequences using standard codon translation tables. Strains were annotated with either their respective clade assignments from the phylogenetic tree or their clinical metadata and analyzed separately. An iterative version of the Metadata-driven Comparative Analysis Tool for Sequences (Meta-CATS) algorithm was then applied to identify amino acid positions showing statistically-significant skewing in the residue distributions either between phylogenetic clades or clinical metadata [30]. The resulting amino acid positions in the nonstructural and structural polyproteins were converted to mature peptide positions using the ViPR database (www.viprbrc.org) [31]. The publicly available E1-E2 heterodimeric structure for the CHIKV E1-E2 proteins was downloaded from the RCSB (PDB: 3J2W) [32, 33]. UCSF Chimera software was used to navigate and visualize the three-dimensional protein structure (PDB: 3J2W) [34].

### Deep-sequencing analysis

Deep sequencing analysis was performed on all sequences to identify the minor variants in the sequence population. This involved generating a consensus sequence from all sequence reads for each sample using the CLC mapping assembly program *(clc_ref_assemble_long)* and then mapping all high-quality trimmed reads sequenced from the sample against its own consensus sequence. The annotated consensus was used to provide biological context and to determine whether the minor variation resulted in a synonymous or a nonsynonymous change, and whether any nonsynonymous changes were categorized as either conservative or non-conservative. To find statistically-significant variations in the population, all forward and reverse reads covering each position were checked and a statistical model using a binomial distribution was generated to ensure that coverage at each base position was above a specific threshold (3%) with a 95% confidence interval, followed by Bonferroni correction for multiple-hypothesis testing. Positions where insufficient coverage existed to provide 95% confidence of a minor allele resulted in the position not being reported in the output. The significant variations existing in the sequencing reads were then included in an automated report against the consensus sequence of the same strain to obtain the percentage of minor alleles in the population.

## Results

### High-throughput sequencing of the complete CHIKV genome

Samples for this study from the three collections are described in Materials and Methods. For the 108 samples obtained from studies in Nicaragua, final assemblies resulted in an average of 20,953 reads per sample, with an average coverage of 348X (range: 60X-1,079X). Final assembly of 67 complete genomes from the strains provided by the Florida BPHL indicated an average of 15,662 reads per sample, with an average coverage of 271X (range: 43X-745X). From the NYSDOH, sequencing of 10 strains resulted in an average of 14,559 reads per sample, with an average coverage of 254X (range: 180X-513X). Together, these new complete genome sequences substantially increase the number of complete genome sequences available in GenBank from the Asian genotype and allow for more in-depth sequence analyses.

### Characteristics of complete CHIKV genomes

In order to maximize the reliability of downstream phylogenetic analyses, our first step was to identify and remove any sequences that displayed evidence of recombination. RDP results suggested that one of 456 coding sequences, CHIKV_STMWG02, displayed evidence of potential recombination and was consequently excluded from further phylogenetic analysis. The CHIKV genome was highly conserved across all samples, with an average nucleotide similarity of 96.20% and an average amino acid similarity of 98.24%. Nonstructural genes (NSP1, NSP2, NSP3, and NSP4) showed similar levels of heterogeneity as structural genes (Capsid, E3, E2, 6K, and E1), with the average nucleotide similarity ranging from 95.66% (NSP3) to 96.74% (NSP2), and the average amino acid similarity ranging from 95.51% (6K) to 99.18% (NSP2). NSP2 was the most conserved gene across the genome as a whole. We then wanted to gain a better understanding of the role that selection pressure played in the sequence diversity within this collection. As such, we investigated the aligned sequences for individual amino acid sites under diversifying selection (positive selection) by three methods (see Materials and Methods), using a significance level of p<0.05. Nonstructural protein NSP1 site 148 was identified as being under diversifying selection by all three methods, whereas no codons in the structural polyprotein were detected to be under diversifying selection by more than one method.

### Epidemiological history of CHIKV Asian genotype and CO clade

**Fig 1:**
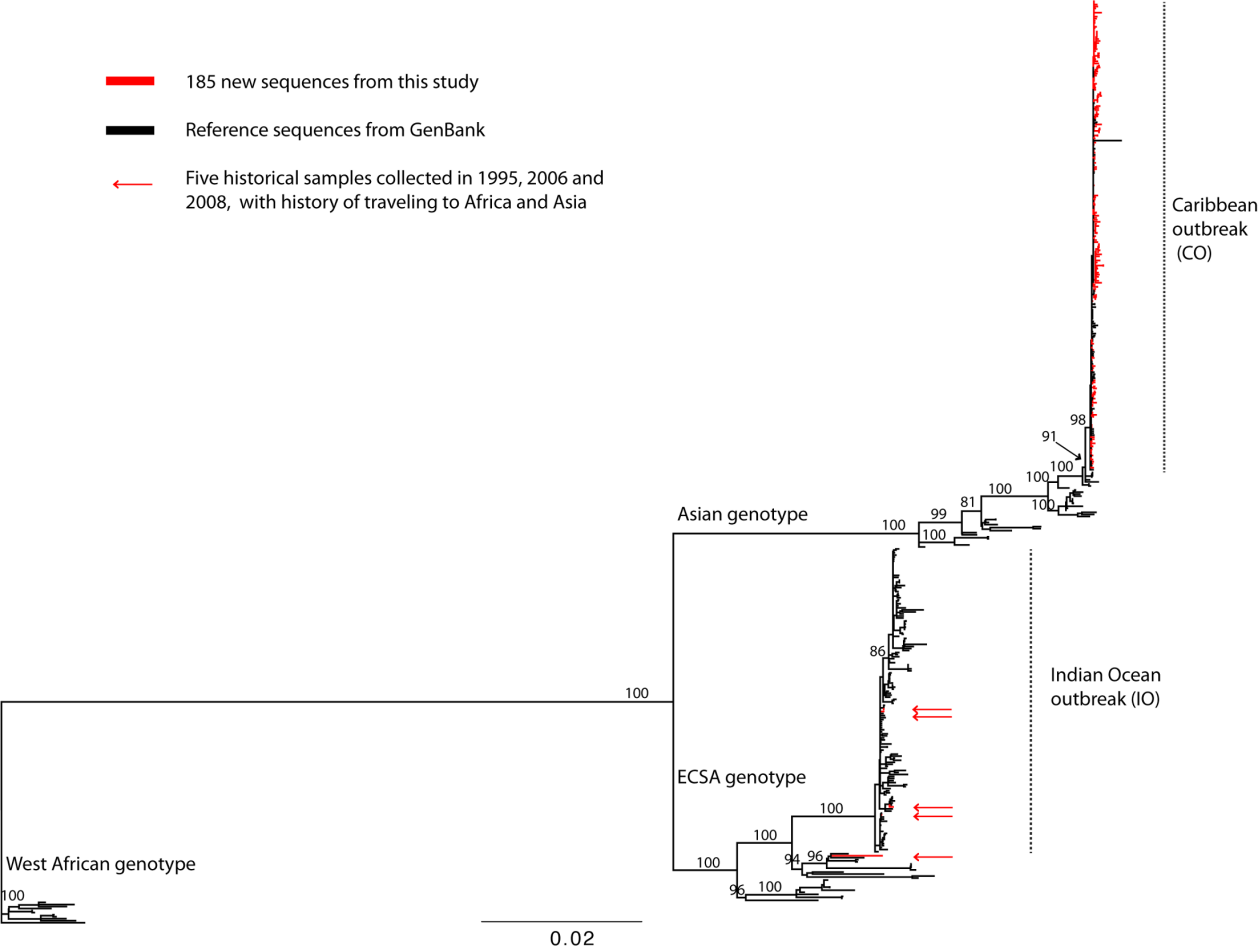
Maximum likelihood phylogenetic tree of complete coding nucleotide sequences of CHIKV. Well-supported nodes by bootstrap values over 70% are marked in the tree. Three genotypes (West African, ECSA, and Asian) and clades (Indian Ocean and Caribbean outbreaks) are described in the trees. New sequences sampled from the Americas are colored in red. The phylogenetic tree is midpoint-rooted, and the scale bar represents the number of nucleotide substitutions per site. Arrows indicate known recent travel of the human hosts to Africa or Asia in the ECSA genotype.

**Fig 2:**
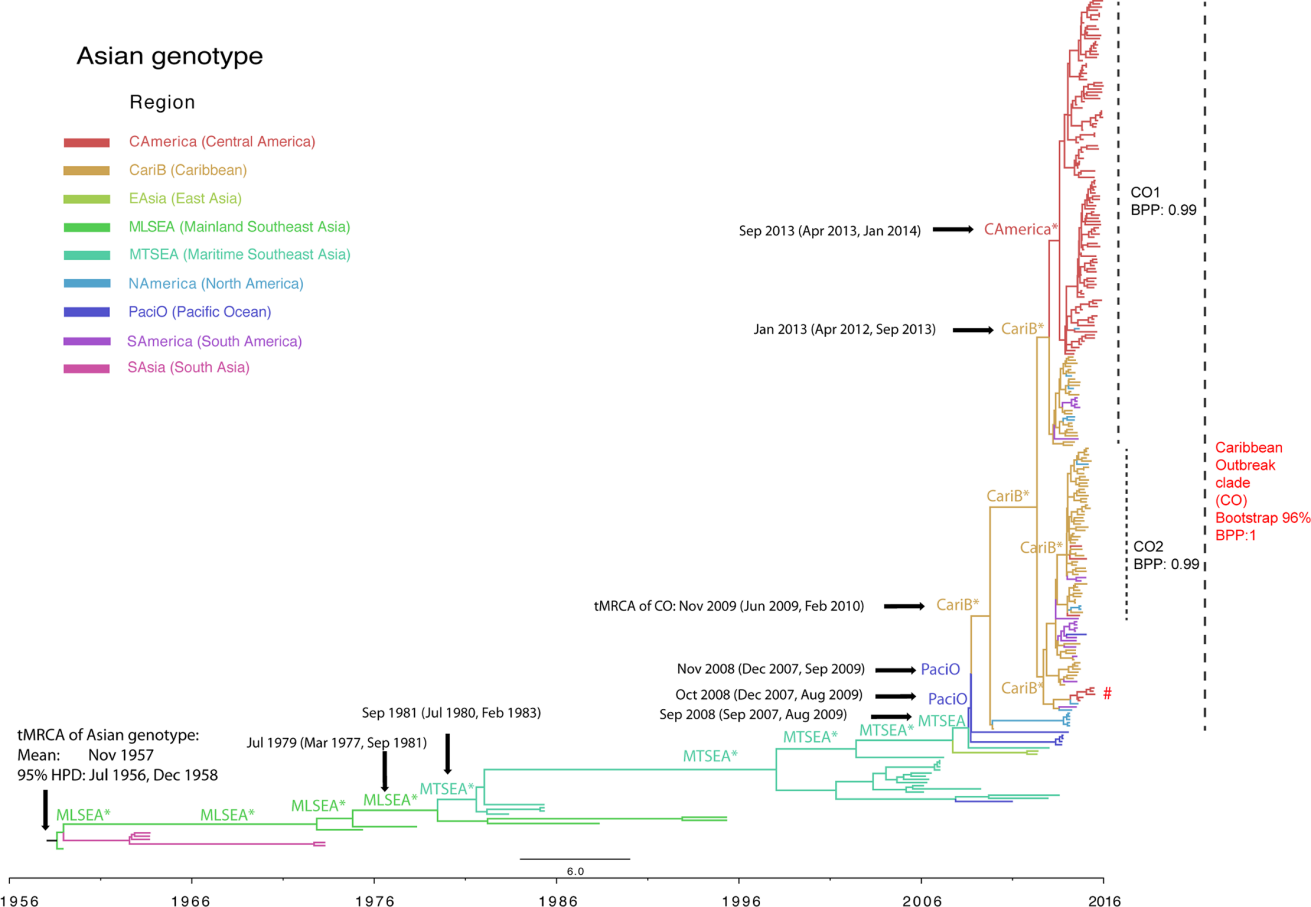
Phylodynamics of CHIKV Asian genotype epidemics. Time-scaled maximum clade credibility (MCC) tree of the CHIKV Asian genotype was inferred by Bayesian analysis. Sequences from different regions are colored as described in the key. The Caribbean outbreak (CO) clade is supported by Bayesian posterior probabilities (BPP) as 1 in BMCMC analysis and bootstrap values 96% in the maximum likelihood phylogeny. Subclades CO1 and CO2, supported by BPP over 0.98, are also marked in the tree. A small group of Nicaraguan sequences that are relatively distinct from other Nicaraguan sequences are highlighted by a red hash symbol (#). The major ancestral geographic states (state probabilities over 0.5) at the backbone of phylogenetic tree are colored by region and labeled in the tree. The nodes with state probabilities greater than 0.9 are marked by asterisks. The mean and 95% highest posterior density (HPD) of the most recent common ancestor (tMRCA) of the entire Asian genotype and tMRCAs of virus movement between regions are also indicated. The scale bar represents chronological time (in years).

To gain a better understanding of the evolutionary relationships between our collection of CHIKV sequences and those that had previously been generated, we constructed a maximum likelihood phylogenetic tree of all available sequences (Fig 1). This global CHIKV phylogeny revealed the presence of the three major genotypes of CHIKV West African, ECSA, and Asian genotypes that have spread around the globe, consistent with previous studies [19, 35, 36]. The recent Indian Ocean outbreak (IO) and Caribbean outbreak (CO) form monophyletic clades that are descendants of the ECSA and Asian genotypes, respectively. Our newly-sequenced strains collected from patients in Nicaragua, Florida (with recent travel history to the Caribbean), and New York after 2014 (also with recent travel history to the Caribbean) fell into the CO clade. Four historical strains collected from New York during 2006–2008 (with recent travel history to India, La Reunion, and Sri Lanka) fell into the IO clade, while one strain collected from a patient in New York in 1995 (with travel history to Central Africa Republic) belongs to the ECSA genotype but not the IO clade (Fig 1). The long branch lengths of the ECSA and Asian genotypes in the CHIKV global phylogeny suggest that they have been circulating in Africa and Asia for many years prior to being detected or sampled. We analyzed the Asian (Fig 2) and ECSA genotypes (S1 Fig) separately to better understand their evolutionary dynamics and to more accurately reconstruct their epidemic history.

We then reconstructed a time-scaled MCC phylogenetic tree focusing on the Asian subtree to provide a finer level of granularity regarding the relationships between historical strains within the Asian genotype and the sequences isolated during the Caribbean/Americas epidemic (Fig 2). This tree provides estimates of temporal signal, with the CO clade being well-supported by a Bayesian posterior possibility (BPP) of 1 and a bootstrap value of 96% in a maximum likelihood tree. The mean evolutionary rate of the entire Asian genotype was estimated to be 4.33E-4 substitutions/site/year, with a 95% highest posterior density (HPD) between 3.54E-4 and 5.15E-4 substitutions/site/year. The time to the most recent common ancestor (tMRCA) of the Asian genotype was estimated as November 1957, with a 95% HPD between July 1956 and December 1958.

**Fig 3:**
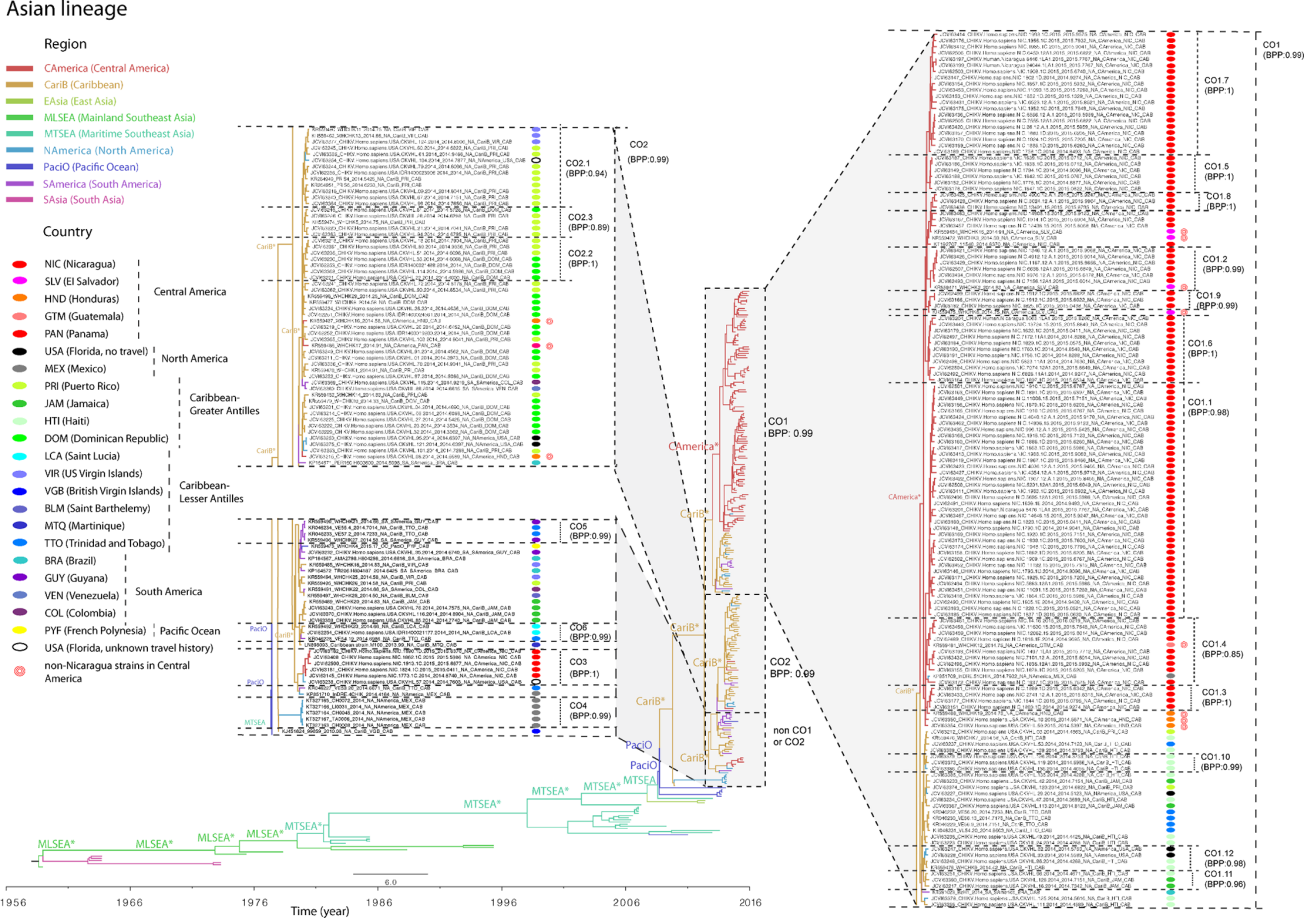
Phylogeographic clustering of Caribbean/Americas epidemic CHIKV strains. The Bayesian maximum clade credibility (MCC) tree of CHIKV Asian genotype was annotated to reflect the geographic origin of samples collected in the Americas. The CO clade was split into three parts, magnified, and indicated by gray shading: subclade CO1 (right side of the tree), subclade CO2 (left middle panel), and non-CO1/CO2 strains (left lower panel). Sequences from different regions are colored in branches as described in the key. The original countries of infections (either indigenous or travel-related) are depicted with colored ovals next to the complete strain names and described in the key as well.

Our analysis suggests that the Asian genotype has circulated in mainland southeast Asia since the 1950s and then migrated to the surrounding islands around 1980 (state probabilities over 0.9, marked by asterisks in Fig 2). These viruses continued to spread across the Pacific (state probabilities over 0.5 but less than 0.9) and Caribbean islands (state probabilities over 0.9, marked by asterisks) from 2008–2009 prior to causing the epidemic in the Western hemisphere in 2014–2015. Since 2013, CHIKV continued to spread from the Caribbean islands to Florida in North America; Brazil, Colombia, and Guyana in South America; as well as Nicaragua and other Central American nations (Table 1, Fig 2, Fig 3). However, the limited number of non-CO strains prohibited us from accurately modeling the virus movements between southeast Asia, the Pacific islands, and the Caribbean.

### Genetic diversity of epidemic strains in Caribbean/Americas (CO clade) and different epidemiological dynamics of CHIKV in Nicaragua and Florida

With the rapid explosion of strains that contributed to the Caribbean/Americas epidemic, we did not observe significant subclades within the CO clade in the maximum likelihood phylogenetic tree. However, we observed considerable genetic diversity in the BMCMC phylogenetic tree, which enabled the separation of two large subclades (CO1 and CO2, BPP>0.9, Fig 3), a few small subclades (CO3-CO6, BPP>0.9, Fig 3), and some monophyletic groups within the CO1 and CO2 subclades (CO1.1-CO1.12 and CO2.1-CO2.3, Fig 3). Sequences from Nicaragua and Florida showed different epidemiological dynamics. Nicaraguan sequences tended to cluster together in the upper portion of the CO clade but did not form a single subclade. The exception was a small group of Nicaraguan sequences (marked by the red pound sign in Fig 2) that were phylogenetically distinct from the other Nicaraguan sequences (Fig 2 and Fig 3). This suggests several introductions and rapid local spread of the viruses in Nicaragua during the 2014–2015 epidemic. In contrast, the CHIKV epidemic in Florida was characterized by multiple introductions of the viruses from travelers (imported cases) but very limited autochthonous transmission. Sequences from Florida are located in the lower portion of the CO clade and did not form a single subclade either. With the travel information that was available, the Florida sequences were found to be phylogenetically proximal to the sequences from the regions in Caribbean to which the cases reported travel (see the following section, Fig 3).

### Phylogeographic clustering of Caribbean/Americas epidemic strains

We next wanted to determine how the topology of the phylogenetic tree consisting of sequences collected during the Caribbean/Americas epidemic (CO clade) was influenced by the geographical location of the patient. The availability of recent travel history for each patient in the majority of the newly-generated sequences from the United States enabled us to trace the origin of the infection. To determine the phylogeographic structure of the CHIKV CO clade, we performed phylogeny-trait association (BaTS) tests on the phylogenies of the CO clade alone. This analysis revealed significant phylogenetic clustering by geographical location (p-values for AI and PS < 0.001), suggesting stronger spatial clustering of the virus by either sampling country or country of recent travel than expected by chance alone. To enable a more in-depth view of how geographical location of isolation correlated with tree topology, the CO clade from the Bayesian MCC tree of Asian genotype sequences was annotated with the country of origin (Fig 3). Superimposing the geographical location information on the tree for the CO strains revealed strong evidence that the strains primarily cluster together by the geographical origin of the infection (Fig 3). The branches of the tree were colored based on the regions where the samples were isolated, while the ovals represent the probable origins of infections. In general, strains isolated from the same place tended to cluster together (Fig 3). Our newly sequenced strains from New York and Florida with travel history cluster with sequences of strains that were originally isolated from the same geographical location (Fig 3). Five Florida strains with no travel history (black ovals in Fig 3) show the local transmission of CHIKV in Florida.

Almost all Nicaraguan strains fall into the CO1 subclade, with the exception of a monophyletic group of five sequences in the CO3 subclade (Fig 3, left lower panel), and Nicaraguan strains in the CO1 clade clustered with strains from other countries in Central America (e.g., El Salvador, Guatemala, and Honduras, highlighted with red concentric circles in Fig 3, right panel). The rest of the CO1 strains are from countries in the Caribbean Greater Antilles (Puerto Rico, Haiti, and Jamaica), Caribbean Lesser Antilles (Trinidad and Tobago), and North America (Florida and Mexico), indicating the viral movements between the Caribbean and North America. Although Puerto Rico and the Dominican Republic within the Caribbean Greater Antilles were the most common sources of the origin of infection for sequences belonging to the CO2 subclade (Fig 3, left upper panel), limited strains were isolated from Caribbean Lesser Antilles (US Virgin Islands), Central America (Honduras and Panama), South America (Colombia, Venezuela, and Brazil), and North America (Florida). The rest of the non-CO1/CO2 sequences (Fig 3, left lower panel) have more diverse origins than the CO1 and CO2 sequences with representative sequences from North America (Mexico), South America (Guyana, Brazil, Colombia), Caribbean Greater Antilles (Puerto Rico, Jamaica), Caribbean Lesser Antilles (Trinidad and Tobago, US Virgin Islands, Saint Barthelemy, Saint Lucia, Martinique, British Virgin Islands), and Central America (Nicaragua). Overall, our phylogeographic analysis across multiple collections suggests numerous international transmission events.

### Amino acid substitutions of Caribbean/Americas epidemic strains

**Fig 4:**
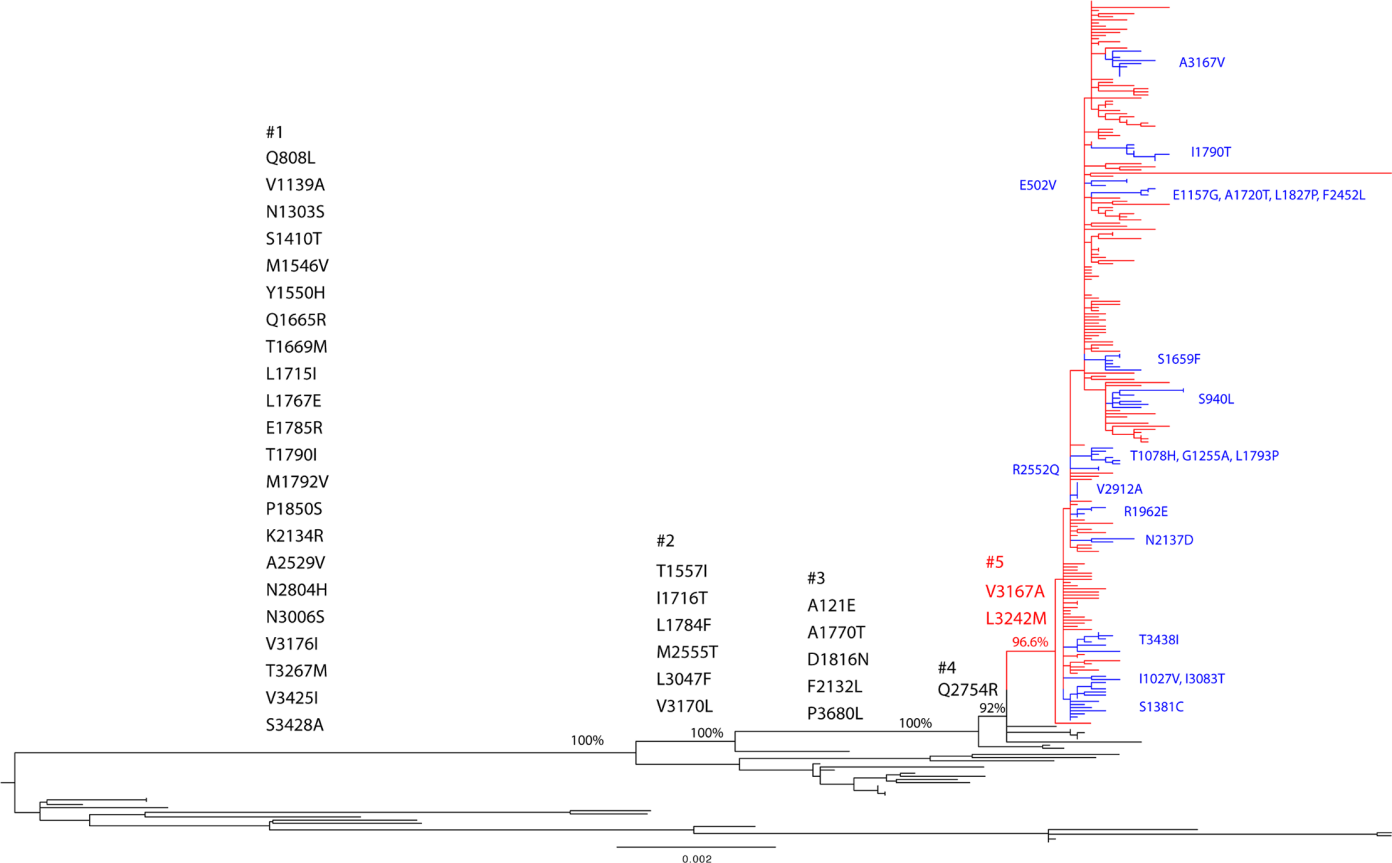
Maximum likelihood tree of CHIKV Asian genotype. The major nodes with bootstrap values above 70% are indicated. The CO clade, supported by bootstrap value 96.6%, is colored in red. Amino acid substitutions that contribute to clade diversity are mapped to the branches of the tree. CO-clade-specific amino acid substitutions are marked in red. Monophyletic groups within CO clade and associated amino acid changes are marked in blue. The scale bar represents genetic distance.

Amino acid substitutions that have become fixed into the evolutionary history of the CHIKV Asian genotype were mapped to the branches of maximum likelihood phylogeny (Fig 4). The CO clade is characterized by amino acid substitutions V3167A and L3242M (Fig 4, internal branch 5), relative to the recent Asian strains (after the year 2000), which have all the amino acid changes on internal branches 1 to 4 (Fig 4). Additional amino acid substitutions for small monophyletic groups within the CO clade were also mapped to the maximum likelihood tree (Fig 4). Given that the topology of the BMCMC phylogeny showed subclades and monophyletic groups within the CO clade (Fig 3), we performed statistical comparisons of the amino acid residues that significantly differed between these subclades and monophyletic groups using Meta-CATS. Specifically, we compared sequences belonging to CO1 vs. CO2, CO2.1 vs. CO2.2, and CO1.1 vs. CO1.4 vs. CO1.6 vs. CO1.7 (Fig 3). Clades CO1.2, CO1.3, and CO1.5 were excluded from these statistical comparisons because of insufficient numbers of sequences. We then compared the results from the maximum likelihood phylogeny to those from the BMCMC phylogeny.

Significant amino acid substitutions suggested by the Meta-CATS analysis for BMCMC phylogeny were consistent with those suggested by the maximum likelihood phylogeny (Table 2, Fig 4). We identified 2 positions that showed strict residue separation between the CO clades, based on the consensus sequence. Aligned position 1381 in the nonstructural polyprotein, corresponding to amino acid position 48 in the NSP3 mature peptide, consists solely of serine residues in the CO1 and CO2.2 clades; however, this serine is replaced by a cysteine residue in all 13 strains belonging to clade CO2.1. Similarly, at aligned position 3438 in the structural polyprotein, located at position 155 of the E1 mature protein, only threonine residues are observed in clades CO1 and CO2.1, whereas CO2.2 strains code exclusively for an isoleucine. In addition, significant variation was detected at nonstructural polyprotein positions 1157, 1720, 1827, and 2452 of CO1.4 strains when compared to CO1.1, CO1.6, and CO1.7. Specifically, 3 of 9 residues differ in the CO1.4 strains when compared to the other 3 clades. Interestingly, these 3 strains (NIC.7101, NIC.1497, and NIC.1835) were all isolated from Nicaragua in 2015 and belong to a sub-clade of CO1.4 (Fig 3 and Fig 4).

**Table 2:** Significant residue diversity between CO clades.

Polyprotein Name	Compared Clades	Polyprotein Position	Mature Peptide Position^a^	P-value	Residue Diversity (Clade 1)	Residue Diversity (Clade 2)	Residue Diversity (Clade 3)	Residue Diversity (Clade 4)
nsp	CO1 vs CO2	1381^b^	NSP3, 48	1.39E-08	142 S	13 C, 42 S		
	CO2.1 vs CO2.2	1381	NSP3, 48	6.89E-05	13 C	7 S		
	CO1.1 vs CO1.4 vs CO1.6 vs CO1.7	940	NSP2, 405	5.68E-05	38 S	9 S	11 S	7 L, 13 S
		1157	NSP2, 622	2.63E-05	38 E	6 E, 3 G	11 E	20 E
		1720	NSP3, 387	2.63E-05	38 A	6 A, 3 T	11 A	20 A
		1827	NSP3, 494	2.63E-05	38 L	6 L, 3 P	11 L	20 L
		2452	NSP4, 589	2.63E-05	38 F	6 F, 3 L	11 F	20 F
sp	CO1 vs CO2	2984^c^	E2, 185	0.03109	142 S	3 C, 52 S		
		3438	E1, 155	9.63E-05	142 T	7 I, 48 T		
	CO2.1 vs CO2.2	3438	E1, 155	6.89E-05	13 T	7 I		
	CO1.1 vs CO1.4 vs CO1.6 vs CO1.7	3167	E2, 368	0.01345	29 A, 9 V	9 A	11 A	20 A

^a^Position in RefSeq strain S27, GenBank Accession: NC_004162.

^b^Underlined positions indicate significant differences between multiple clades.

^c^We did not find this site in maximum likelihood phylogeny.

### Structural analysis of significant positions

**Fig 5:**
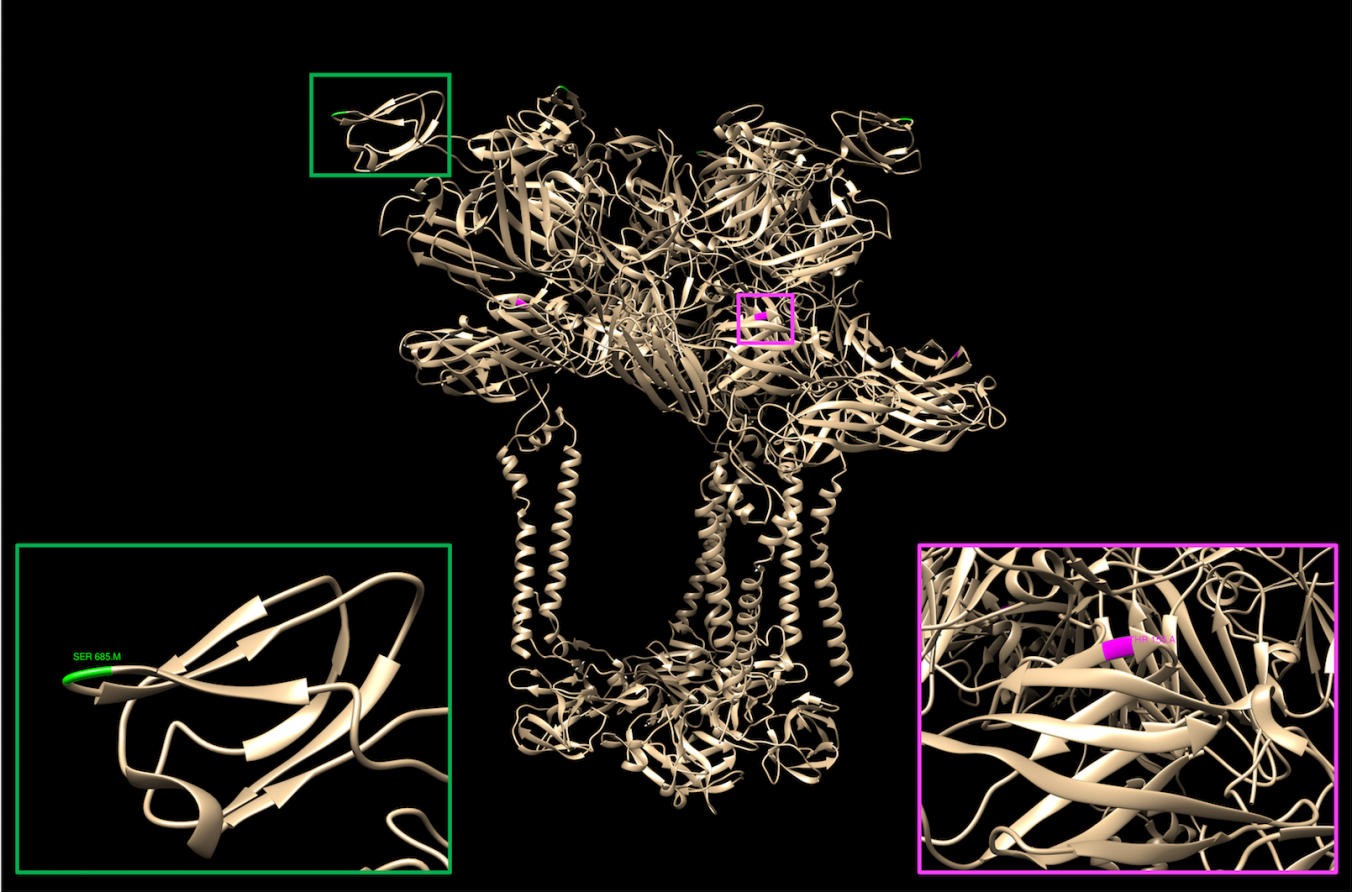
Structural analysis of clade-specific amino acid residues. Threonine 155 in the E1 protein (magenta) and Serine 185 in the E2 protein (green) were visualized on the three-dimensional hetero-multimeric structure (PDB: 3J2W). The colored insets show a magnified view of the regions surrounding T155 and S185 (magenta and green, respectively).

We subsequently visualized the amino acid differences identified by Meta-CATS that were located in the structural E1 and E2 proteins onto an existing three-dimensional protein structure (3J2W) to better understand the contribution of the significant amino acid variation that was observed. This structure includes substantial regions of the E1 and E2 proteins that have been co-crystallized in a conformation that has been modeled into a complete virion structure. We focused specifically on the residues that differed in the E1 and E2 regions, since in general they have a higher likelihood of affecting the host B-cell immune response and entry into target cells. While residues 368 and 371 in the E2 protein were found to significantly differ between the CO and previously existing Asian strains, these residues were not resolved in the available crystal structure [33]. However, threonine 155 in E1 and serine 185 in E2 were both present in visible regions of the structure (Fig 5). Threonine 155 in E1 is located within a pocket that is formed from the quaternary structures of E1 and E2. In contrast, serine 185 in E2 is located at the tip of a small globular domain that extends outward from the surface of the virion.

### Deep sequencing and minor variant analysis

With an average sequencing depth of at least 250X across all three CHIKV collections, we re-examined the read information to identify minor variants that were present as intra-host quasispecies within each clinical sample. Deep sequencing analysis of the nonstructural polyprotein region identified 250 minor variants that met our filtering criteria in strains belonging to at least one of the three collections and 56 minor variants that were observed across strains belonging to 2 or more collections. In the structural polyprotein, there were 156 minor variants present in strains belonging to at least one collection, with 30 of these variants detected across at least 2 collections (S1–S3 Tables).

We identified 20 codons in the nonstructural polyprotein that contained statistically significant minor variation across all three virus collections including: 25, 26, 29, 30, 158, 163, 164, 235, 320, 376, 377, 1059, 1060, 1234, 1358, 1853, 1976, 2173, 2204, and 2293. Codon numbering was used to facilitate cross-referencing between minor variants that affect amino acid sequence and other analyses. To determine whether any of the detected minor variants were present within nonsynonymous codons identified by Meta-CATS as significantly differing between subclades, we cross-referenced the 2 sets of results. Codon 1381 in the nonstructural polyprotein had detectable minor variation only within the New York collection. Interestingly, the consensus sequence in this codon codes for serine in members of the CO1 subclade but codes for either serine or cysteine in the CO2 subclade, with minor variants coding for either amino acid being detected. Similarly, 12 codons in the structural polyprotein were found to have minor variants across all three collections at positions: 20, 22, 24, 510, 511, 769, 805, 810, 889, 944, 948, and 1135. These findings suggest that dominant variants have emerged within the viral genome while genetic diversity is still being maintained at the population level as quasispecies that contain multiple minor variants.

### Correlation of demographic, clinical and geographic features with viral genetic variation in Nicaraguan sequences

Clinical symptoms, disease severity, and related metadata fields were collected as part of the Nicaraguan study. Specifically, host demographic information was available for all 108 Nicaraguan samples, and clinical information was available for 95 of these samples. We performed two phylogeny-trait association statistics (AI and PS) to identify whether Nicaraguan CHIKV strains might exhibit some phylogenetic clustering by host demographics and/or disease severity (Table 3). We found no significant association of age distribution and sex ratio with viral genetic variation (i.e., phylogenetic topology) in Nicaraguan strains. Hence, phylogenetic clustering by age and sex was not greater than that expected by chance alone. However, the analysis revealed significant phylogenetic clustering of Nicaraguan strains by the presence of dehydration and myalgia separately, based on both clustering statistics, AI and PS (p-value ≤ 0.004) (Table 3). Additionally, significant phylogenetic clustering of Nicaraguan strains by only one statistical method was found for rash (AI p-value = 0.036) and arthralgia (PS p-value = 0.013) (Table 3).

**Table 3:** Results of the phylogeny-trait association tests for demographic and clinical characteristics of chikungunya patients in Nicaragua.

Comparison^a^	Statistic^b^	Observed value ^c^	Null value ^c^	*P* ^d^
Sex of patient	AI	5.20 (4.27–6.11)	5.31 (4.27–6.29)	0.42
Male: 38/108	PS	30.09 (28.0–32.0)	31.48 (28.25–34.21)	0.20
Age of patient	AI	8.32 (7.40–9.26)	8.02 (6.94–9.08)	0.67
<5 years: 28/108	PS	52.17 (50.0–55.0)	51.12 (47.05–54.78)	0.64
6–10 years: 32/108				
11–18 years: 45/108				
>18 years: 3/108				
Dehydration: 29/95	AI	3.01 (2.29–3.73)	5.04 (4.15–5.89)	**<0.001**
	PS	22.08 (20.0–24.0)	29.65 (26.99–32.0)	**<0.001**
Headache: 43/95	AI	4.01 (3.21–4.81)	5.04 (4.02–5.98)	0.05
	PS	28.43 (26.0–31.0)	31.49 (27.93–34.59)	0.05
Vomiting: 22/95	AI	3.16 (2.46–3.88)	3.58 (2.79–4.34)	0.17
	PS	20.13 (19.0–21.0)	20.04 (18.23–21.45)	0.43
Myalgia: 49/95	AI	3.67 (2.85–4.47)	5.60 (4.62–6.53)	**<0.001**
	PS	28.65 (26.0–31.0)	34.84 (31.33–37.96)	**0.004**
Rash: 24/95	AI	2.89 (2.21–3.59)	3083 (3.02–4.58)	**0.036**
	PS	19.77 (18.0–21.0)	21.59 (19.35–23.23)	0.1
Abdominal pain: 10/95	AI	2.19 (1.66–2.72)	1.87 (1.34–2.40)	0.85
	PS	9.99 (10.0–10.0)	9.69 (8.82–10.0)	1
Arthralgia: 76/95	AI	2.53 (1.92–3.15)	3.22 (2.46–3.94)	0.07
	PS	15.15 (14.0–16.0)	17.56 (15.93–18.72)	**0.013**
Retro-orbital pain: 10/95	AI	1.56 (1.02–2.13)	1.87 (1.36–2.38)	0.15
	PS	9.96 (10.0–10.0)	9.67 (8.82–10.0)	1
Fever: 93/95	AI	0.71 (0.67–0.75)	0.41 (0.18–0.64)	0.99
	PS	2.0 (2.0–2.0)	1.99 (2.0–2.0)	1

^a^No. of cases/total No. of patients

^b^AI, association index; PS, parsimony score.

^c^Value with 95% confidence interval.

^d^Statistically significant results are in bold.

Based on the tree topology, we hypothesized that samples from particular geographic areas within Nicaragua could be enriched in virus strains assigned to different phylogenetic subclades. To test this hypothesis, we applied a hypergeometric statistical test. While the majority of the sequenced isolates were collected in Managua, statistically significant results were observed for CO1.1 strains in Masaya (3/3 virus samples in this subclade and location; p-value 0.041), and subclade CO1.4 in 2 separate locations including Managua (5/8 virus samples in this subclade; p-value = 0.047) and Nueva Segovia (2/8 virus samples in this subclade; p-value = 0.014).

## Discussion

This work presents a large number of newly-acquired coding-complete CHIKV sequences collected from patients in the United States and Nicaragua during the 2014–2015 Caribbean and Latin American epidemic. The new data significantly increase the number of publicly-available CHIKV sequences, especially of the Asian genotype, and consequently allowed a comprehensive study of the evolution and genetic diversity of the CHIKV Asian genotype in the Americas. We reconstructed the epidemic history of the Asian genotype and estimated the mean evolutionary rate as 4.33E-4 substitutions/site/year (95% HPD: 3.54E-4–5.15E-4 substitutions/site/year). Our phylogenetic analyses revealed considerable genetic diversity and phylogeographic clustering within the CO clade and described different epidemiological dynamics of CHIKV in the Americas. Specifically, the Nicaragua epidemic was characterized by several introductions and rapid local spread of the virus, while the Florida epidemic was characterized by multiple introductions but limited autochthonous transmission. Arbovirus transmission by Aedes sp. mosquitoes in Florida has been well documented [37], especially related to DENV transmission [38–40]. We also identified the significant amino acid substitutions within the entire Asian genotype as well as within the CO clade. Additionally, our deep sequencing minor variants analysis investigated intra-host diversity of CHIKV strains in the CO clade, especially regarding the nonsynonymous codons identified previously by phylogenetic reconstructions. Finally, we found significant phylogenetic clustering according to the presence of dehydration and myalgia (separately) in the Nicaraguan sequences.

The genetic analyses suggest that CHIKV is a conserved virus, with over 95% nucleotide/amino acid similarity across the genome. Previous studies based on partial genomes and individual genes yielded similar results [35]. Codon 148 in the nsp1 coding region was identified as undergoing positive selection pressure. Given that this position is located in a nonstructural protein and is not present in any currently known B-cell epitopes, it is possible that this residue plays a role in modulating intracellular interactions and/or the host T-cell immune response. Insertion/deletion regions in the CHIKV 3’UTR are associated with genotype/subtype classification and most likely occurred as a founder effect due to their adaptation to mosquitoes [41]. Since insertion/deletion does not count as “substitution” in viral evolution, we did not include 3’UTR region in our BEAST analysis. The substitution rate of CHIKV was estimated to range from 4E-4 (Asian genotype) to 6E-4 (ECSA genotype) substitutions/site/year by our temporal phylogenetic analysis, consistent with previous studies based on fewer numbers of CHIKV genome sequences [19, 42]. The substitution rate is lower than human influenza A virus (3E-3 substitutions/site/year) and Zika virus (1E-3 substitutions/site/year) [43, 44], but similar to other RNA viruses, such as human respiratory syncytial virus A (5E-4 substitutions/site/year) [45], West Nile virus (5E-4 substitutions/site/year) [46], and Ebola virus (8E-4 substitutions/site/year) [47].

Epidemic history of the CHIKV Asian genotype reconstructed by phylogenetic analyses showed a significant temporal-spatial evolutionary pattern, especially among non-CO strains, and indicated that the CHIKV Asian genotype diverged about 50–60 years ago. Long internal branches of non-CO sequences in the phylogenetic trees indicated extensive genetic diversity and suggested that the non-CO viruses have been circulating for many years prior to being detected or sampled. Significant geographical clustering of non-CO strains reflected limited viral transmission between regions, with a previous study showing similar results [19]. In contrast, due to the rapid spread and explosive transmission of the CO viruses, the CO clade showed distinct bush-like patterns with several polytomies. Furthermore, there was more viral transmission between different regions in the CO clade than in non-CO strains. A previous study suggested an intertwined transmission network among different countries and regions [19]. However, with the large number of newly-acquired sequences during the 2014–2015 Caribbean/Americas epidemic, the CO clade presents significant phylogeographic clustering as well. In particular, our Florida sequences with recent travel history available enabled robust interpretations regarding distinct virus introductions and co-circulating virus strains in different regions. However, a lack of such metadata for many previously-published sequences may prevent us from gaining a complete picture of international virus transmission. In Nicaragua, the geographic enrichment analysis revealed a higher-than-expected presence of strains belonging to the CO1.4 subclade in both Managua and Nueva Segovia, which are located relatively far apart (~300 km = 186 miles). This finding could indicate either co-circulation or separate introductions of this virus subclade into two geographic regions.

The large sampling of sequences from Nicaragua and Florida in this study allowed us to investigate the epidemiological dynamics of the CHIKV Asian genotype in these two regions during the 2014–2015 epidemic. Different strains of the viruses were introduced to the Americas, including Nicaragua, and were the cause of most of the outbreaks in different regions. However, although there were multiple introductions of the virus in Florida, there was limited autochthonous transmission of CHIKV during the 2014–2015 Caribbean/Americas epidemic. Florida has a large population of residents that travel to countries with ongoing transmission and a high number of visitors that were prior residents of these countries. A few possible reasons for the lack of sustained transmission in Florida could exist. First, the climate of Florida and the mosquito vector population may not be optimal to create the same environment as in other countries in the Caribbean to sustain transmission for a long period of time. Second, the U.S. and Florida have better arbovirus surveillance systems, prevention methods (for example, home window screens, air conditioning, regular garbage collection services, use of repellent, promotion of dumping standing water around the home, and promotion of covering mosquito breeding sites such as boats, etc.), and mosquito control (through the use of larvicide and adulticide). These factors likely contribute to the lack of ongoing CHIKV transmission in Florida so far.

Next, we investigated the significant amino acid substitutions in the Asian genotype phylogenies and discovered two CO-clade-specific amino acid substitutions, V3167A (E2 V368A) and L3242M (6K L20M), and other amino acid changes within CO clade. A recent comparison of the nonsynonymous amino acid changes that differentiate American and Asian genotype strains also identified these two sites [42]. The amino acid differences that we identified as being significantly different between Asian pre-CO and Asian CO sequences are of interest and could merit additional experimentation to determine whether they are accompanied by a change in phenotype. While we expect that a subset of these positions are consistent among intra-clade strains due to the evolutionary relatedness of these strains, we also expect a small number of these positions to confer some evolutionary advantage, which would explain their persistence in the virus population. Although high specificity of clade-specific residues was not observed at all significantly different positions, such comparisons may identify regions of viral proteins where amino acid changes are tolerated and may play a role in viral replication, fitness, host interaction, or the immune response.

In addition, deep sequencing analysis, which was previously performed by Stapleford *et al*. on a collection of CHIKV strains, identified a large number of minor variants that met the established criteria of having a significant p-value and being present in at least 20 percent of reads for a given position [36]. Interestingly, when we compared the positions on our list to those reported in the previous study [36], we found five positions that matched previous results. Specifically, codons 134 in nsp1, 613 in nsp2, and 116, 345, and 441 in nsp4 overlapped between our study and the prior publication. This finding provides independent verification of quasispecies circulating during the Caribbean outbreak that contained these minor variants, and may indicate convergent evolution among these viruses as they follow a similar, yet independent, evolutionary “path”. Additional laboratory work is needed to determine whether the circulating quasispecies that include minor variants modulate viral replication, host-pathogen interactions, or overall fitness and whether genetic influences, such as covariation, play a role in their dominance.

The location of the two clade-specific variations on the E1-E2 three-dimensional protein structure also provides additional knowledge. The region surrounding threonine 155 is not likely exposed to the host immune system since it is buried in the tertiary and quaternary protein structure and is not present in or near any reported B cell epitopes from the Immune Epitope Database (www.iedb.org) [48]. Conversely, the globular domain surrounding serine 185 contains a linear 18-mer B cell epitope that is located immediately adjacent to S185 [49, 50] and may also play a role in binding to host proteins.

Additional CHIKV sequence data are critical to understand the genetic diversity of the virus and to successfully monitor the epidemic potential of CHIKV in the Americas. Similarly, metadata describing the virus isolation, patient characteristics, and clinical information are also useful in efforts to identify how viral evolution may contribute to changes in transmission, virulence, and/or disease severity. An example that illustrates this point comes from previous CHIKV studies of the 2005–2006 outbreak in La Reunion, which identified a single mutation in the E1 glycoprotein (A226V of the ECSA genotype) [51, 52]. This mutation enhances the fitness of CHIKV in its mosquito vector *Aedes albopictus*, which is more prevalent than *Aedes aegypti* in the southeastern United States and other regions.

In our study, while analyzing whether significant phylogenetic clustering of multiple clinical signs and symptoms was present, we observed that both dehydration and myalgia are well-supported by two statistical methods. Conversely, a high degree of confidence in the significant findings for both rash and arthralgia are less warranted since they were each classified as significant by only one statistical method. Even so, the ability to identify significant clustering of these clinical signs and symptoms was only made possible by the extensive collection of clinical metadata that accompanied patient specimens. The identification of two amino acid substitutions that were associated with headache and the one amino acid difference that was associated with dehydration were found to be significant, although the expected residue specificity was lower than expected. Additional experimentation with these positions is needed to better understand how the virus sequence affects the host response to infection.

In summary, our study reported a large number of CHIKV complete genome sequences collected from multiple regions in the Americas and revealed the epidemiological and evolutionary dynamics of the CHIKV Asian genotype (particularly the CO clade) during the recent CHIKV Caribbean/Latin American epidemic. These results provide additional insight into the evolution of CHIKV during a geographically-diverse epidemic and may have important implications for the control and prevention of other mosquito-borne viruses in the Americas, such as Zika, dengue, and West Nile viruses.

## Supporting information

S1 Fig Phylodynamics of CHIKV ECSA genotype epidemics. Time-scaled maximum clade credibility (MCC) tree of CHIKV ECSA genotype was inferred by Bayesian analysis. Sequences from different regions are colored as described in the key. Indian Ocean clade (IO) and subclades (IO1, IO2, IO3, and IO4) are marked. Five new sequences with travel history to Africa and Asia are labeled by red rectangles next to the relevant branches. The major nodes with posterior probabilities over 0.9 in BMCMC analysis and bootstrap values over 70% in ML phylogeny are indicated in the tree. The major ancestral geographic states (state probabilities over 0.5) at the backbone of phylogenetic tree are colored by region and labelled. The nodes with state probabilities over 0.9 are marked by asterisks. The most recent common ancestor (tMRCA) of entire ECSA genotype and tMRCAs of virus movement between regions are also indicated in the trees. The scale bar represents chronological time (in years).
(TIF)

S1 Table Summary of codons in the nonstructural polyprotein that contain significant minor variants. (DOCX)

S2 Table Summary of codons in the structural polyprotein that contain significant minor variants. (DOCX)

S3 Table Overlap with previous deep-sequencing results [36]. (DOCX)
