# Supplementary figures and images for: Differing epidemiological dynamics of Chikungunya virus in the Americas during the 2014-2015 epidemic
Source: PLoS Negl Trop Dis. 2018 Jul 30;12(7):e0006670. doi: 10.1371/journal.pntd.0006670 (PMC6085065; doi:10.1371/journal.pntd.0006670)

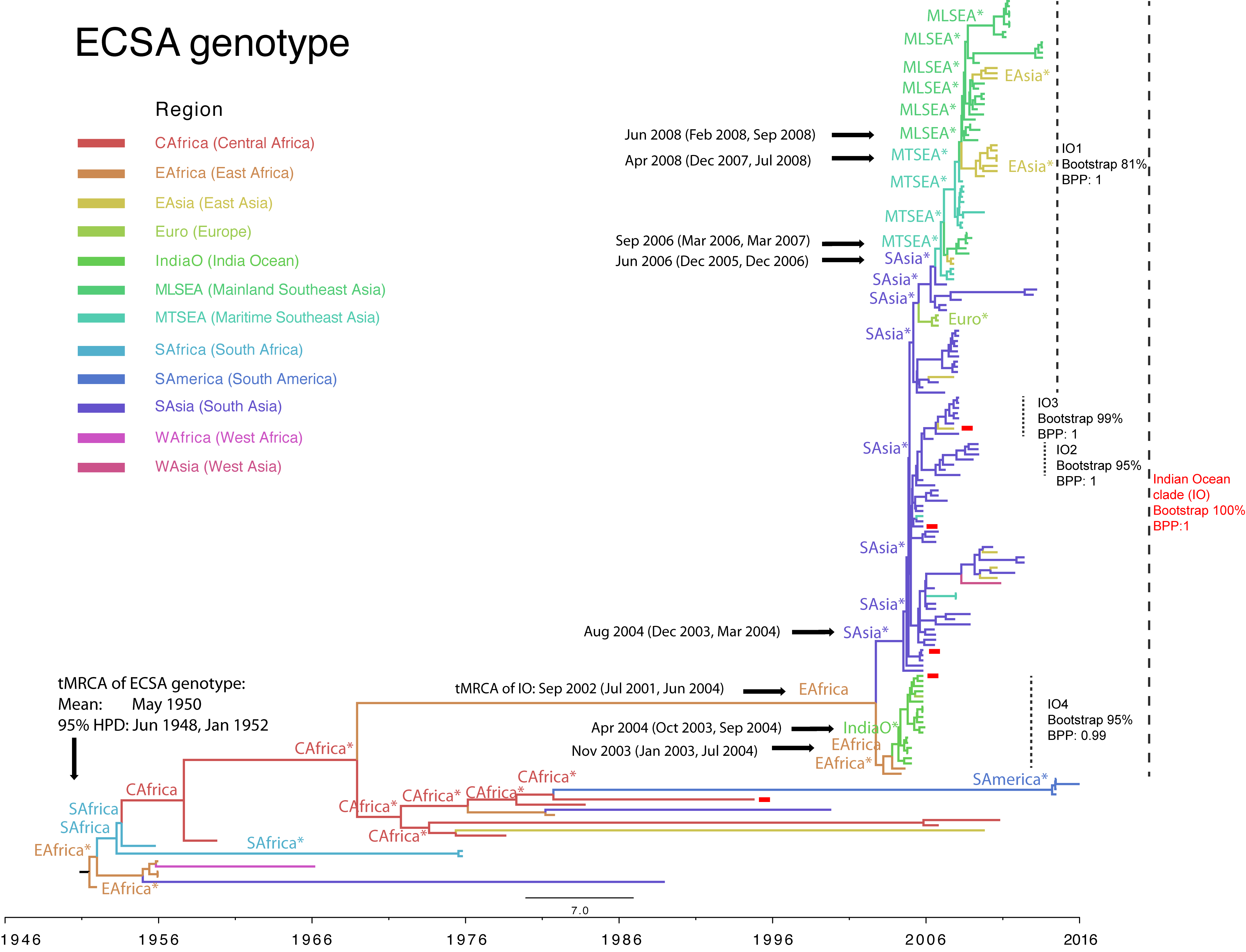

Supplement: S1 Fig — Time-scaled maximum clade credibility (MCC) tree of CHIKV ECSA genotype was inferred by Bayesian analysis. Sequences from different regions are colored as described in the key. Indian Ocean clade (IO) and subclades (IO1, IO2, IO3, and IO4) are marked. Five new sequences with travel history to Africa and Asia are labeled by red rectangles next to the relevant branches. The major nodes with posterior probabilities over 0.9 in BMCMC analysis and bootstrap values over 70% in ML phylogeny are indicated in the tree. The major ancestral geographic states (state probabilities over 0.5) at the backbone of phylogenetic tree are colored by region and labelled. The nodes with state probabilities over 0.9 are marked by asterisks. The most recent common ancestor (tMRCA) of entire ECSA genotype and tMRCAs of virus movement between regions are also indicated in the trees. The scale bar represents chronological time (in years). (TIF) [file pntd.0006670.s001.tif]
