# Supplementary material for: Differing epidemiological dynamics of Chikungunya virus in the Americas during the 2014-2015 epidemic
Source: PLoS Negl Trop Dis. 2018 Jul 30;12(7):e0006670. doi: 10.1371/journal.pntd.0006670 (PMC6085065; doi:10.1371/journal.pntd.0006670)
Supplement: S1 Table — (DOCX) [file pntd.0006670.s002.docx]

**S1 Table. Summary of codons in the nonstructural polyprotein that contain significant minor variants.**

| **NSP Codon Number** | **% samples w/ codon variation (New York)*** | **% samples w/ codon variation (Florida)*** | **% samples w/ codon variation (Nicaragua)*** |
| --- | --- | --- | --- |
| 25 | 9.09% | 4.48% | 34.58% |
| 26 | 9.09% | 7.46% | 37.38% |
| 29 | 9.09% | 5.97% | 37.38% |
| 30 | 9.09% | 10.45% | 42.06% |
| 57 |  |  | 0.93% |
| 85 |  | 2.99% | 0.93% |
| 93 |  | 1.49% |  |
| 104 |  |  | 4.67% |
| 117 |  | 1.49% |  |
| 124 |  |  | 0.93% |
| 125 |  |  | 0.93% |
| 127 |  | 1.49% | 0.93% |
| 134 |  | 4.48% | 1.87% |
| 135 |  | 68.66% | 71.03% |
| 140 |  | 1.49% | 0.93% |
| 141 |  | 13.43% | 13.08% |
| 157 | 9.09% |  | 0.93% |
| 158 | 45.45% | 94.03% | 74.77% |
| 162 | 9.09% |  |  |
| 163 | 9.09% | 8.96% | 0.93% |
| 164 | 36.36% | 10.45% | 0.93% |
| 171 | 18.18% |  |  |
| 188 |  | 4.48% | 0.93% |
| 192 |  |  | 0.93% |
| 228 |  |  | 0.93% |
| 235 | 18.18% | 52.24% | 41.12% |
| 237 |  | 4.48% | 4.67% |
| 238 | 9.09% |  |  |
| 265 | 9.09% |  |  |
| 274 |  |  | 7.48% |
| 275 |  |  | 5.61% |
| 283 |  | 1.49% |  |
| 295 |  |  | 0.93% |
| 300 |  | 1.49% |  |
| 308 |  |  | 0.93% |
| 314 | 9.09% |  |  |
| 318 |  |  | 0.93% |
| 320 | 9.09% | 1.49% | 0.93% |
| 321 |  | 1.49% | 0.93% |
| 326 | 9.09% |  |  |
| 375 |  | 41.79% | 13.08% |
| 376 | 9.09% | 50.75% | 68.22% |
| 377 | 9.09% | 55.22% | 68.22% |
| 386 |  |  | 0.93% |
| 399 |  |  | 0.93% |
| 401 | 9.09% |  |  |
| 403 | 9.09% |  |  |
| 404 | 9.09% |  |  |
| 405 | 9.09% |  |  |
| 406 |  | 1.49% |  |
| 419 |  |  | 0.93% |
| 428 |  | 1.49% | 0.93% |
| 429 |  |  | 0.93% |
| 431 |  |  | 0.93% |
| 453 | 9.09% |  |  |
| 458 | 9.09% | 1.49% |  |
| 507 |  |  | 6.54% |
| 519 |  |  | 0.93% |
| 539 |  | 1.49% |  |
| 551 |  |  | 0.93% |
| 590 |  | 1.49% |  |
| 610 |  |  | 0.93% |
| 641 |  |  | 0.93% |
| 677 |  | 1.49% |  |
| 692 |  | 1.49% |  |
| 765 |  | 1.49% |  |
| 784 | 9.09% |  |  |
| 794 |  |  | 0.93% |
| 795 |  |  | 0.93% |
| 820 |  |  | 0.93% |
| 823 |  |  | 0.93% |
| 853 | 9.09% |  |  |
| 856 |  | 1.49% |  |
| 895 |  |  | 1.87% |
| 911 |  |  | 7.48% |
| 915 |  |  | 6.54% |
| 916 |  | 7.46% | 23.36% |
| 966 |  |  | 1.87% |
| 968 |  | 4.48% | 0.93% |
| 974 |  | 2.99% | 0.93% |
| 980 |  | 1.49% | 0.93% |
| 983 |  |  | 0.93% |
| 1047 |  |  | 0.93% |
| 1059 | 18.18% | 49.25% | 59.81% |
| 1060 | 9.09% | 1.49% | 0.93% |
| 1066 |  | 1.49% |  |
| 1090 |  | 1.49% |  |
| 1108 |  |  | 0.93% |
| 1126 |  |  | 0.93% |
| 1127 |  |  | 0.93% |
| 1128 |  |  | 0.93% |
| 1133 |  | 1.49% |  |
| 1148 |  | 1.49% |  |
| 1179 |  | 25.37% | 14.02% |
| 1192 | 9.09% |  |  |
| 1214 |  | 1.49% |  |
| 1218 |  | 1.49% |  |
| 1233 |  |  | 0.93% |
| 1234 | 18.18% | 70.15% | 54.21% |
| 1238 |  | 1.49% |  |
| 1256 |  |  | 0.93% |
| 1265 |  |  | 0.93% |
| 1266 |  |  | 0.93% |
| 1303 |  | 1.49% |  |
| 1312 | 18.18% |  |  |
| 1329 |  | 1.49% |  |
| 1342 |  | 1.49% |  |
| 1353 |  |  | 0.93% |
| 1358 | 9.09% | 5.97% | 2.80% |
| 1364 | 9.09% |  |  |
| 1381 | 9.09% |  |  |
| 1391 |  |  | 0.93% |
| 1399 |  |  | 0.93% |
| 1410 |  |  | 0.93% |
| 1419 |  | 1.49% | 11.21% |
| 1450 | 27.27% |  |  |
| 1467 |  |  | 0.93% |
| 1470 | 9.09% |  |  |
| 1474 |  | 1.49% |  |
| 1476 |  | 1.49% |  |
| 1490 |  |  | 0.93% |
| 1491 |  | 1.49% |  |
| 1492 |  |  | 0.93% |
| 1493 |  |  | 0.93% |
| 1519 |  | 1.49% |  |
| 1521 |  |  | 1.87% |
| 1528 |  | 1.49% | 0.93% |
| 1537 |  | 1.49% |  |
| 1542 | 9.09% |  |  |
| 1546 | 9.09% |  |  |
| 1550 |  | 1.49% | 0.93% |
| 1552 |  |  | 0.93% |
| 1565 |  |  | 0.93% |
| 1579 | 9.09% |  |  |
| 1586 |  |  | 0.93% |
| 1591 |  | 1.49% |  |
| 1607 |  | 1.49% | 0.93% |
| 1617 |  |  | 0.93% |
| 1625 |  |  | 0.93% |
| 1627 |  |  | 0.93% |
| 1647 | 9.09% |  |  |
| 1651 |  |  | 0.93% |
| 1659 |  |  | 0.93% |
| 1669 |  |  | 0.93% |
| 1680 |  | 1.49% | 0.93% |
| 1697 |  |  | 0.93% |
| 1719 |  |  | 0.93% |
| 1726 |  | 1.49% | 0.93% |
| 1729 |  | 1.49% |  |
| 1742 |  | 1.49% | 6.54% |
| 1747 |  | 1.49% |  |
| 1763 |  | 2.99% |  |
| 1764 |  |  | 0.93% |
| 1766 |  | 1.49% |  |
| 1774 |  | 16.42% | 32.71% |
| 1783 |  | 1.49% |  |
| 1786 |  |  | 0.93% |
| 1812 |  |  | 0.93% |
| 1822 |  |  | 0.93% |
| 1823 |  | 4.48% |  |
| 1824 |  |  | 2.80% |
| 1825 |  |  | 0.93% |
| 1826 |  | 1.49% |  |
| 1829 |  | 1.49% |  |
| 1837 |  |  | 0.93% |
| 1839 | 9.09% |  |  |
| 1842 |  |  | 0.93% |
| 1846 |  | 1.49% |  |
| 1847 |  |  | 0.93% |
| 1853 | 45.45% | 8.96% | 4.67% |
| 1856 |  | 1.49% | 11.21% |
| 1857 | 18.18% |  |  |
| 1868 |  |  | 0.93% |
| 1879 |  |  | 0.93% |
| 1880 |  |  | 1.87% |
| 1887 |  |  | 0.93% |
| 1895 |  |  | 0.93% |
| 1899 | 9.09% |  |  |
| 1911 |  |  | 0.93% |
| 1912 |  |  | 0.93% |
| 1926 |  | 1.49% |  |
| 1929 |  | 1.49% |  |
| 1930 |  |  | 0.93% |
| 1932 |  |  | 0.93% |
| 1934 | 9.09% |  |  |
| 1937 |  | 1.49% |  |
| 1940 |  | 1.49% |  |
| 1943 |  |  | 0.93% |
| 1945 |  | 1.49% |  |
| 1962 |  |  | 3.74% |
| 1963 |  |  | 11.21% |
| 1964 |  |  | 0.93% |
| 1971 |  |  | 0.93% |
| 1975 |  | 1.49% | 0.93% |
| 1976 | 9.09% | 1.49% | 0.93% |
| 1997 |  | 1.49% |  |
| 2010 |  | 11.94% | 7.48% |
| 2023 |  |  | 0.93% |
| 2039 | 9.09% |  |  |
| 2051 |  | 1.49% |  |
| 2053 |  |  | 0.93% |
| 2059 |  |  | 0.93% |
| 2080 |  |  | 0.93% |
| 2087 |  |  | 0.93% |
| 2089 |  | 1.49% |  |
| 2093 |  |  | 0.93% |
| 2095 |  | 1.49% |  |
| 2098 |  |  | 0.93% |
| 2118 |  | 1.49% |  |
| 2119 | 9.09% |  |  |
| 2121 |  |  | 0.93% |
| 2133 |  |  | 0.93% |
| 2147 |  |  | 0.93% |
| 2148 |  | 1.49% |  |
| 2162 |  |  | 0.93% |
| 2164 |  |  | 0.93% |
| 2168 |  | 16.42% | 14.95% |
| 2173 | 45.45% | 100.00% | 80.37% |
| 2177 | 45.45% |  | 0.93% |
| 2203 |  |  | 0.93% |
| 2204 | 27.27% | 1.49% | 1.87% |
| 2208 | 36.36% |  |  |
| 2216 |  |  | 0.93% |
| 2223 |  |  | 1.87% |
| 2224 |  |  | 0.93% |
| 2233 |  | 1.49% |  |
| 2241 |  |  | 1.87% |
| 2242 |  |  | 1.87% |
| 2260 |  | 1.49% |  |
| 2278 |  | 1.49% |  |
| 2280 |  | 1.49% |  |
| 2293 | 45.45% | 100.00% | 93.46% |
| 2297 | 45.45% |  |  |
| 2299 |  | 10.45% | 19.63% |
| 2300 |  | 8.96% | 2.80% |
| 2306 |  |  | 0.93% |
| 2330 |  |  | 0.93% |
| 2337 |  |  | 0.93% |
| 2350 |  |  | 0.93% |
| 2354 |  | 1.49% |  |
| 2387 |  | 1.49% |  |
| 2395 |  |  | 0.93% |
| 2402 |  |  | 10.28% |
| 2403 |  |  | 10.28% |
| 2416 |  |  | 0.93% |
| 2421 |  | 1.49% | 0.93% |
| 2437 |  | 43.28% | 54.21% |
| 2440 |  | 5.97% | 11.21% |
| 2453 |  | 1.49% |  |
| 2471 |  |  | 0.93% |

* % samples w/ codon variation was calculated by dividing the number of sequences that contained significant minor variants at each respective codon in each collection by the number of sequences in the respective collection.
