# Supplementary material for: Differing epidemiological dynamics of Chikungunya virus in the Americas during the 2014-2015 epidemic
Source: PLoS Negl Trop Dis. 2018 Jul 30;12(7):e0006670. doi: 10.1371/journal.pntd.0006670 (PMC6085065; doi:10.1371/journal.pntd.0006670)
Supplement: S2 Table — (DOCX) [file pntd.0006670.s003.docx]

**S2 Table. Summary of codons in the structural polyprotein that contain significant minor variants.**

| SP Codon Number | % samples w/ codon variation (New York) | % samples w/ codon variation (Florida) | % samples w/ codon variation (Nicaragua) |
| --- | --- | --- | --- |
| 3 |  |  | 0.93% |
| 20 | 45.45% | 83.58% | 75.70% |
| 22 | 45.45% | 80.60% | 73.83% |
| 23 |  | 1.49% | 0.93% |
| 24 | 18.18% | 47.76% | 52.34% |
| 27 |  |  | 19.63% |
| 31 |  | 7.46% | 2.80% |
| 44 |  | 1.49% |  |
| 77 |  |  | 0.93% |
| 80 |  | 2.99% |  |
| 87 | 9.09% |  |  |
| 88 |  |  | 0.93% |
| 90 |  | 2.99% |  |
| 93 |  | 1.49% |  |
| 95 |  |  | 0.93% |
| 102 |  |  | 0.93% |
| 104 |  |  | 0.93% |
| 105 |  |  | 0.93% |
| 119 |  | 7.46% | 1.87% |
| 120 |  |  | 1.87% |
| 172 |  |  | 0.93% |
| 173 |  | 1.49% |  |
| 182 |  | 1.49% |  |
| 202 |  | 1.49% |  |
| 208 |  | 4.48% | 2.80% |
| 213 |  | 1.49% |  |
| 221 |  | 1.49% | 0.93% |
| 230 |  | 1.49% |  |
| 240 |  |  | 0.93% |
| 254 |  |  | 0.93% |
| 255 |  | 1.49% |  |
| 262 | 9.09% |  |  |
| 266 |  |  | 0.93% |
| 278 |  | 1.49% |  |
| 280 |  | 1.49% |  |
| 287 |  | 1.49% |  |
| 315 |  | 2.99% | 3.74% |
| 316 |  |  | 0.93% |
| 317 |  | 2.99% | 2.80% |
| 319 |  | 1.49% | 0.93% |
| 323 | 9.09% |  |  |
| 324 | 9.09% |  |  |
| 329 | 9.09% |  |  |
| 361 |  | 1.49% |  |
| 365 |  |  | 0.93% |
| 368 |  |  | 0.93% |
| 371 |  |  | 0.93% |
| 373 |  |  | 1.87% |
| 394 | 9.09% |  |  |
| 403 |  |  | 0.93% |
| 409 |  | 1.49% | 0.93% |
| 426 |  |  | 0.93% |
| 433 |  | 1.49% |  |
| 434 |  |  | 0.93% |
| 453 |  | 1.49% |  |
| 456 |  |  | 2.80% |
| 457 |  |  | 0.93% |
| 463 |  |  | 0.93% |
| 479 |  |  | 0.93% |
| 484 | 9.09% |  |  |
| 498 |  | 1.49% |  |
| 499 |  |  | 0.93% |
| 510 | 36.36% | 32.84% | 44.86% |
| 511 | 18.18% | 16.42% | 21.50% |
| 522 |  |  | 0.93% |
| 552 |  | 5.97% | 0.93% |
| 583 | 9.09% |  |  |
| 585 |  |  | 9.35% |
| 587 |  |  | 0.93% |
| 589 |  |  | 3.74% |
| 596 |  | 1.49% |  |
| 597 |  |  | 4.67% |
| 604 |  | 2.99% | 3.74% |
| 605 |  |  | 0.93% |
| 611 |  |  | 9.35% |
| 618 |  |  | 4.67% |
| 623 |  |  | 4.67% |
| 628 |  |  | 0.93% |
| 632 |  |  | 0.93% |
| 637 |  |  | 4.67% |
| 641 |  |  | 3.74% |
| 642 |  |  | 3.74% |
| 643 |  |  | 7.48% |
| 644 |  |  | 3.74% |
| 649 |  |  | 4.67% |
| 655 |  |  | 0.93% |
| 656 |  |  | 1.87% |
| 658 |  | 1.49% | 2.80% |
| 660 |  |  | 0.93% |
| 661 |  |  | 1.87% |
| 668 |  |  | 1.87% |
| 674 |  |  | 1.87% |
| 689 |  |  | 1.87% |
| 693 |  |  | 3.74% |
| 696 |  |  | 1.87% |
| 699 |  | 1.49% |  |
| 700 |  |  | 1.87% |
| 705 |  |  | 3.74% |
| 706 |  | 1.49% |  |
| 707 |  |  | 0.93% |
| 708 |  |  | 0.93% |
| 709 |  |  | 1.87% |
| 710 | 36.36% |  | 0.93% |
| 711 |  |  | 0.93% |
| 717 |  |  | 0.93% |
| 756 |  |  | 0.93% |
| 764 |  | 1.49% |  |
| 768 | 9.09% | 1.49% |  |
| 769 | 90.91% | 91.04% | 86.92% |
| 771 |  | 1.49% |  |
| 772 |  |  | 1.87% |
| 784 | 9.09% |  |  |
| 804 |  | 2.99% | 1.87% |
| 805 | 45.45% | 16.42% | 22.43% |
| 808 |  | 1.49% |  |
| 809 |  | 1.49% |  |
| 810 | 72.73% | 38.81% | 16.82% |
| 813 |  |  | 1.87% |
| 855 |  |  | 0.93% |
| 862 |  | 1.49% |  |
| 875 |  | 1.49% |  |
| 881 |  | 1.49% |  |
| 889 | 9.09% | 2.99% | 24.30% |
| 908 |  |  | 0.93% |
| 934 |  |  | 0.93% |
| 943 |  | 1.49% |  |
| 944 | 90.91% | 97.01% | 81.31% |
| 948 | 18.18% | 22.39% | 30.84% |
| 951 |  |  | 0.93% |
| 966 |  |  | 0.93% |
| 982 |  | 1.49% |  |
| 1047 |  |  | 0.93% |
| 1055 |  | 1.49% |  |
| 1057 | 9.09% |  |  |
| 1111 |  |  | 0.93% |
| 1121 |  |  | 1.87% |
| 1122 |  |  | 1.87% |
| 1123 |  | 4.48% | 42.99% |
| 1126 |  | 2.99% | 18.69% |
| 1132 | 9.09% |  |  |
| 1135 | 27.27% | 4.48% | 34.58% |
| 1174 |  |  | 0.93% |
| 1187 |  |  | 1.87% |
| 1190 |  |  | 0.93% |
| 1191 |  |  | 0.93% |
| 1193 |  |  | 0.93% |
| 1195 |  |  | 0.93% |
| 1198 |  |  | 0.93% |
| 1203 |  |  | 0.93% |
| 1209 |  |  | 0.93% |
| 1211 |  |  | 0.93% |
| 1217 |  |  | 0.93% |
| 1226 |  |  | 0.93% |
| 1230 |  |  | 0.93% |
| 1233 | 9.09% |  | 1.87% |
| 1236 |  | 1.49% |  |

* % samples w/ codon variation was calculated by dividing the number of sequences that contained significant minor variants at each respective codon in each collection by the number of sequences in the respective collection.
