# Supplementary material for: Differing epidemiological dynamics of Chikungunya virus in the Americas during the 2014-2015 epidemic
Source: PLoS Negl Trop Dis. 2018 Jul 30;12(7):e0006670. doi: 10.1371/journal.pntd.0006670 (PMC6085065; doi:10.1371/journal.pntd.0006670)
Supplement: S3 Table — (DOCX) [file pntd.0006670.s004.docx]

**S3 Table: Overlap with previous deep-sequencing results [**[**36**](#_ENREF_36)**]**

| Coding Region | Nucleotide position | Reported amino acid position | aa position (polyprotein)* | Cumulative Percentage Overlap w/  Stapleford *et al.* |
| --- | --- | --- | --- | --- |
| nsp1 | 476 | 134 | 134 | 2.703 |
| nsp2 | 3519 | 613 | 1148 | 0.541 |
| nsp3 | 4219 | 48 | 1381 | 0.541 |
| nsp4 | 5999 | 116 | 1975 | 1.081 |
| nsp4 | 6687 | 345 | 2204 | 3.243 |
| nsp4 | 6975 | 441 | 2300 | 4.865 |

*Amino acid positions are mapped to CHIKV/Homo sapiens/SXM/H-20235-STMARTIN-2013/2003 strain (GenBank: KX262991).
